# A novel deletion mutation in *KMT2A* identified in a child with ID/DD and blood eosinophilia

**DOI:** 10.1186/s12881-019-0776-0

**Published:** 2019-03-06

**Authors:** Haixia Zhang, Bingwu Xiang, Hui Chen, Xiang Chen, Tao Cai

**Affiliations:** 1 Second Xiangya Hospital, Central South University, Changsha, 410002 Hunan China; 2 Physical Medicine and Rehabilitation Center, The Second Affiliated Hospital and Yuying Children’s Hospital of Wenzhou Medical University, Zhejiang, China; 3 The Second Affiliated Hospital of Wenzhou Medical University, Zhejiang, China; 4 Experimental Medicine Section, National Institute of Dental and Craniofacial Research, National Institutes of Health, Bethesda, MD 20892 USA

**Keywords:** *KMT2A* mutation, Intellectual disability, Eosinophilia, Whole exome sequencing, Case report

## Abstract

**Background:**

The *KMT2A* gene encoded lysine methyltransferase plays an essential role in regulating gene expression during early development and hematopoiesis. To date, 92 different mutations of *KMT2A* have been curated in the human gene mutation database (HGMD), resulting in Wiedemann-Steiner syndrome (WDSTS) and intellectual disability (ID)/developmental delay (DD).

**Case presentation:**

In this report, we present a de novo heterozygous deletion mutation [c.74delG; p. (Gly26Alafs*2)] in the *KMT2A* gene, which was identified by trio-based whole exome sequencing from a 5.5-year-old boy with ID/DD, stereotypic hand movements and blood eosinophilia. Many deleterious germline mutations of *KMT2A* have been documented to affect development of central nervous system, oral and craniofacial tissues, but not blood eosinophils.

**Conclusions:**

This is the first report of a rare case with ID/DD as well as eosinophilia, resulting from a previously undescribed null mutation of *KMT2A*. Our findings expand the phenotypical spectrum in affected individuals with *KMT2A* mutations, and may shed some insight into the role of KMT2A in eosinophil metabolism.

**Electronic supplementary material:**

The online version of this article (10.1186/s12881-019-0776-0) contains supplementary material, which is available to authorized users.

## Background

Neurodevelopmental disorders are a wide range of developmental brain dysfunctions, such as intellectual disability (ID), developmental delay (DD), autism spectrum disorder (ASD), epilepsy, and attention deficit hyperactivity disorder (ADHD). It has been estimated approximately 2% of children with ID/DD [1]; many of them are resulted from genetical alterations. Recent analysis showed that 42% neurodevelopmental disorders in children are caused by de novo mutations [2]. With the development of high-throughput sequencing technology, such as the whole-exome sequencing (WES), thousands of genes have been identified to be associated with neurodevelopmental disorders [3–6].

Germline mutations in the *KMT2A* gene (OMIM: 159555) were first identified in patients with Wiedemann-Steiner syndrome [7], which is characterized by hypertrichosis cubiti associated with short stature, consistent facial features, mild to moderate ID, behavioral difficulties, and hypertrichosis on the back (WDSTS, OMIM: 605130). On the other hand, many different somatic mutations or chromosomal rearrangements involving the *KMT2A* gene have been identified in affected individuals or families with leukemia, myeloid/lymphoid or mixed-lineage (OMIM: 159555).

In our previous studies, we utilized trio-based whole-exome sequencing (trio-WES) on hundreds of affected individuals with neurodevelopmental diseases, and identified multiple de novo or inherited genetic mutations in different causal genes, such as *ANK3*, *PLA2G6*, *BCL11A*, and *PAK2* [8–11]. In the present study, a de novo deletion mutation in *KMT2A*, which is predicted to be a null allele, is identified in a boy with ID/DD, stereotypic hand movements, and blood eosinophilia.

## Case presentation

**Fig. 1:**
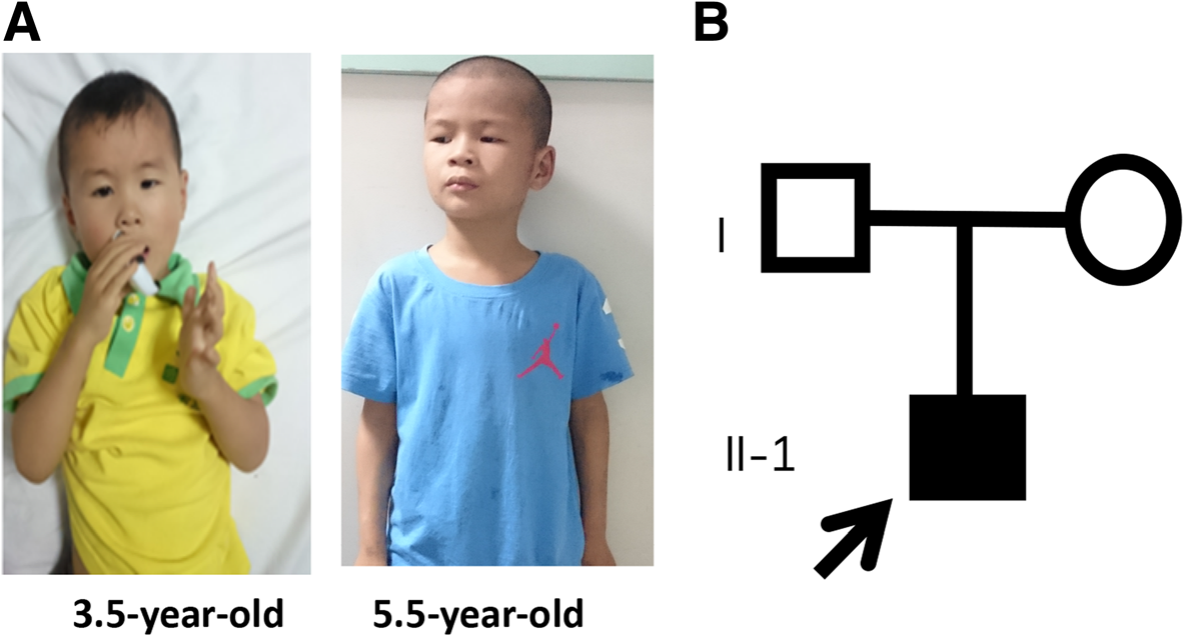
Photographs of the affected boy and the pedigree of his family. (**a**) Facial appearance of the child at 3.5 (left panel) and 5.5 (right panel) years old, respectively. (**b**) The filled square represents the affected proband, empty symbols represent unaffected individuals, square represents male; the circle, female

The proband was from a nonconsanguineous family; his parents were phenotypically normal. He was first referred to the hospital when he was 3.5-year-old (Fig. 1A**,** left panel) with a language developmental delay, a middle level of intellectual disability (ID), and stereotypic hand movements. He was born at 40 weeks in an uneventful spontaneous delivery. His height, body weight, and head circumference at birth were 50.0 cm, 3450 g, and 33 cm respectively, which implies no significant prenatal growth retardation. There were no feeding difficulties and epilepsy. He was able to hold his head up at around 5 months old, sit unaided at 7 months, and walk at 17 months. He started babbling words like “baba”, “mama” around 6 months. At age of 3.5 years, he could only speak a single word, like “yi”, but not a complete sentence. Also, he could not point at an object. He could not communicate with others, and avoided eye-to-eye contact with others. He made repetitive and purposeless movements, like hand waving, tapping and scratching. According to the standard of growth and development of children (0~7-year-old Chinese boy), his growth in height (90 cm, − 3 SD), weight (12 kg, − 2.5 SD) and head circumference (47.5 cm, − 2 SD) was significantly delayed. His score in the infant-junior middle school students social living ability scale (S-M Scale) was low. Physical examination in neurological system and cranial MRI found no obvious abnormalities. At the age of 5.5 years (Fig. 1A, right panel), his height was 102 cm (− 3 SD), weight 15 kg (− 2.5 SD), and head circumference 48.5 cm (− 2 SD).

**Fig. 2:**
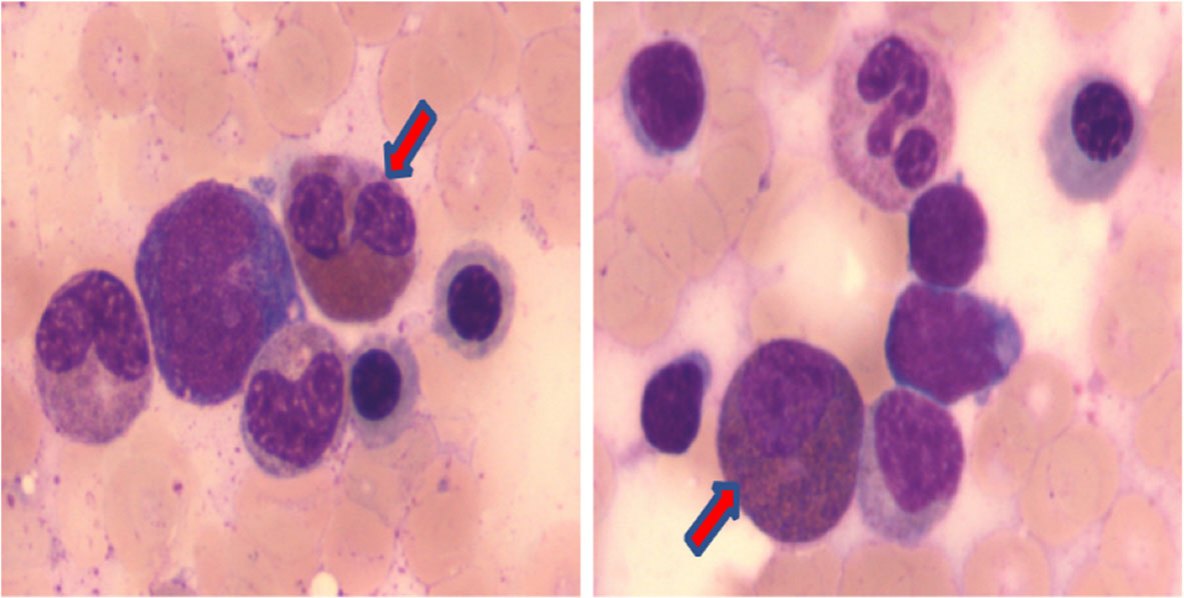
Bone marrow aspirate smear analysis shows eosinophilia. Eosinophils are indicated by arrows

First peripheral venous blood analysis on 3.5-year-old showed increased absolute count of eosinophils (1.847 × 10^9/L, compared to normal level < 0.5*10^9^/L) as well as increased eosinophil ratio (17.1%) [12]. After eight months, his eosinophils numbers and eosinophil ratio still remained at higher levels (1.112 × 10^9/L and 10.8%, respectively). Furthermore, bone marrow puncture analysis showed granulocyte hyperplasia (Fig. 2) and increased eosinophils ratio (5.5%). In addition, blood lymphocyte subset analysis showed no alteration. Virus detection was negative. C-reactive protein and erythrocyte sedimentation rate were normal. Allergy testing was negative. Other clinical data, such as EKG, echocardiogram, chest X-ray, and ultrasonography of abdomen, were all normal.

A trio-based whole exome sequencing (WES) was performed on the affected individual and his parents. After removing low quality readings and adapter or contaminant sequences, 60.8 Mb cleaning readings per person were produced. Further variant analysis was based on Genome Analysis Toolkit (GATK) annotation, which was described in more details in previous studies [9, 13, 14]. Approximately 91.54% of target sequences were covered for more than 10 times. After relatively common variants (MAF ≥ 0.1%) were removed based upon several commonly used databases (i.e., dbSNP142, ExAC, 1000 Genomes and 2000 Chinese Han Exome Sequence database, ChES). Potential inherited and de novo mutations (DNM) related to brain developmental disorders [8, 9] and blood cell development were screened using a recently developed program mirTrios [15]. Deleterious variants were predicted utilizing several commonly used online programs, such as SIFT (http://sift.jcvi.org), Polyphen2 (http://genetics.bwh.harvard.edu/pph2/), MutationTaster (http://www.mutationtaster.org) and PROVEAN (http://provean.jcvi.org/index.php), etc. Sequence variants were interpreted based on ACM standards and guidelines [16].

Finally, bioinformatic analysis identified a de novo mutation of c.74delG in exon 1 of the *KMT2A* gene (GenBank acc. no., NM_001197104.1), which was predicted to result in a premature stop-gain mutation p.(Gly26Alafs*2) in the encoded protein (i.e., histone-lysine N-methyltransferase 2A, GenBank acc. no., NP_001184033.1). Since the mutation is located at the beginning of the *N*-terminal region of the gene product, it is assumed to be a null allele, thereby predicting to be a very strong pathogenic mutation.

**Fig. 3:**
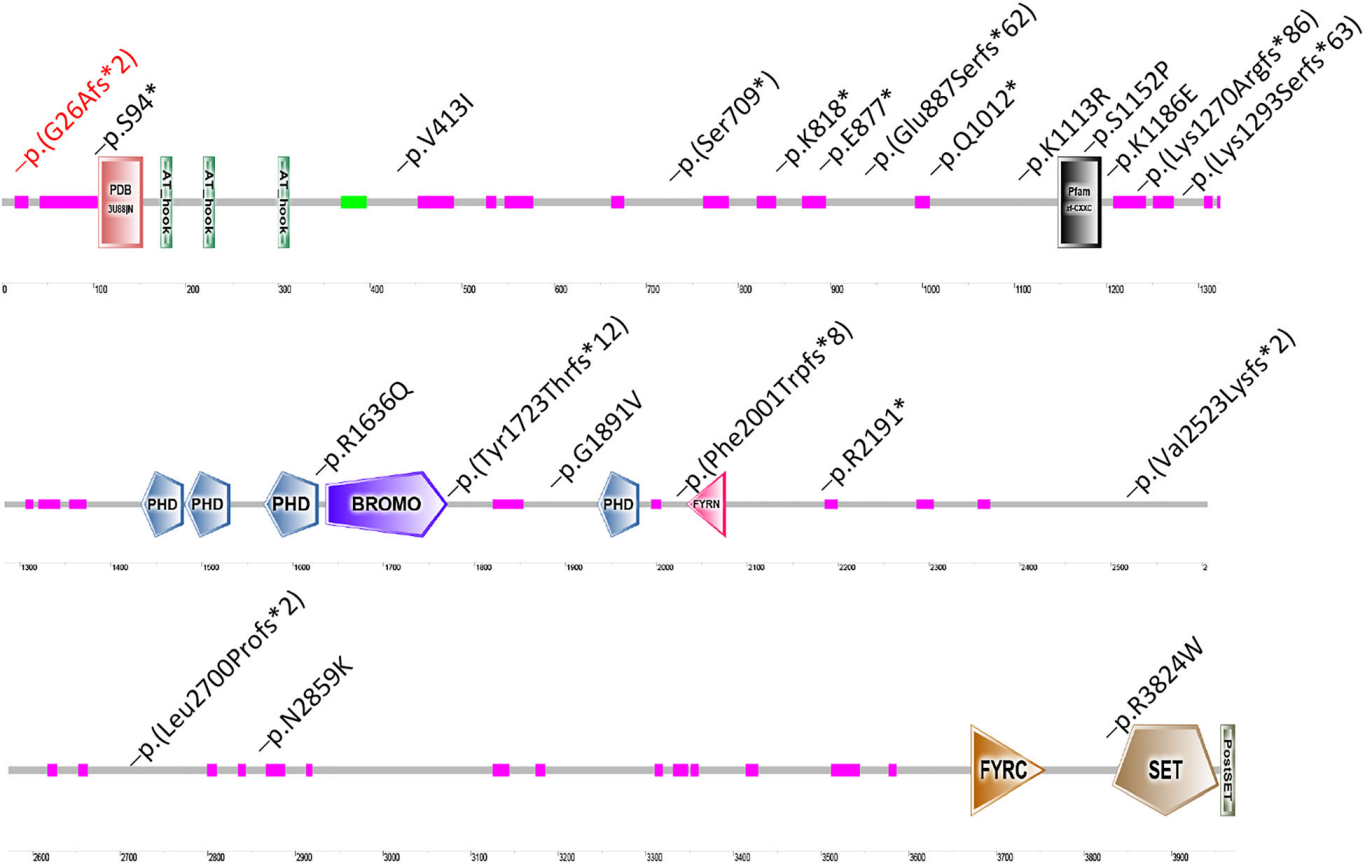
Locations of ID/DD-associated KMT2A mutations. 22 mutations in affected individuals with ID/DD disorders are mapped. Colored boxes depict specific functional domains of the encoded protein using SMART program (http://smart.embl-heidelberg.de/). The mutation in red represents the current case. Four additional mutations with no predicted protein positions are not shown in the diagram

In HGMD database, there are 67 different mutations responsible for Wiedemann-Steiner syndrome (WDSTS), compared to additional 25 mutations for ID/DD (Table 1). Further analysis revealed more nonsense and loss-of-function (LoF) mutations in the affected individuals with WDSTS compared to the ID/DD group, suggesting LoF alleles are implicated in more severe clinical manifestations in WDSTS group. Since the present case mainly showed mild ID and short stature, but not distinctive facial appearance, we compared the genotype of the boy with additional 25 mutations detected in affected individuals with ID/DD in HGMD (Fig. 3). Approximately 68% of the mutations, including non-sense, indel, and splicing alleles, are predicted to be LoF alleles, while eight of them (32%) are hypomorphic missense alleles. However, none of these previously genotyped cases, to the best of our knowledge, were reported with blood eosinophilia.

**Table 1:** *Analysis of KMT2A* mutations in Wiedemann-Steiner syndrome vs ID/DD

	Wiedemann-Steiner syndrome (%)	ID/DD (%)	This study (ID/DD)
Missense	**14 (20.9%)**	8 (32)*	
Nonsense	22 (32.8)*	**5 (20)**	
Splicing	4 (6.0)	2 (8)	
Small deletion	18 (26.9)	8 (32)	1
Small insertion	7 (10.4)	2 (8)	
Gross deletion	2 (2.9)	0 (0)	
**Total LoF alleles**	53 (79.1)*	**17 (68)**	1
**Total mutations**	67	25	1

*, *P* < 0.01

## Discussion and conclusions

The *KMT2A* gene-encoded H3K4 methyltransferase contains multiple conserved functional domains, such as DNA-binding AT-hook motifs, a cysteine-rich CXXC domain, and a C-terminal SET domain (Fig. 3). KMT2A is abundantly expressed in brain, blood and bone marrow and interacts with multiple target genes for transcriptional regulation [17], thereby playing an important role in the development of central nervous system and hematopoietic stem cell differentiation [18, 19].

Different from the effect of germline mutations in *KMT2A* on brain development, somatic truncations or fusion mutations in *KMT2A* are frequently found to be associated with lymphoid leukemia and myeloid leukemia [20–23]. And yet, a germline missense mutation in *KMT2A* also is identified by whole-exome sequencing to segregate with four patients in a family with B-cell lymphoma [24], suggesting a role of KMT2A-encoded protein in hematopoietic stem cell development.

*KMT2A* (a.k.a., *MLL*) is abundantly expressed in lymphoid-derived T cells and myeloid granulocytes like the eosinophils (BioGPS and Human Protein Atlas). Eosinophils are a kind of white blood cell, which is originated from bone marrow hematopoietic stem cells. Blood eosinophilia (> 0.5*10^9^/L) [25] results from overproduction of eosinophils from abnormal myeloid progenitor cells, thereby serving as an early sign of hematological malignancy [26]. Persistent eosinophilia may be caused by chromosomal rearrangements and gene mutations [27]. Somatic mutations in *KMT2A* in patients with leukemia or myeloid/lymphoid or mixed-lineage (OMIM: 159555) may accompany with hypereosinophilia. Interestingly, macrophages with a striated eosinophilic cytoplasm were frequently noted in *Mll*-*AF4* fusion gene knock-in mice [28], an animal model of lymphoid and myeloid deregulation and hematologic malignancy. In addition, mutations in six additional genes are found to be associated with the phenotype “blood eosinophilia” (Additional file 1: Table S1). However, no deleterious mutations in any of these genes are found in the trio-whole-exome analysis of the present case.

In summary, we identified a previously undescribed de novo stop-gain mutation in *KMT2A* in a boy with ID/DD as well as persistent blood eosinophilia. Whether the eosinophilia is directly resulted from the *KMT2A* null mutation needs to be validated in further studies, preferably in additional cases with loss-of function mutations in the *KMT2A* gene.

## Additional file

Additional file 1: **Table S1.** Human Blood Eosinophilia-associated Mutated Genes (DOCX 26 kb)

## Abbreviations

ADHD: Attention deficit hyperactivity disorder
AML-M5: Acute monoblastic leukemia
AMML-M4: Myelomonocytic leukemia
ASD: Autism spectrum disorder
DD: Developmental delay
H3K4: Histone H3 lysine 4
HGMD: Human gene mutation database
ID: Intellectual disability
KMT2A: Lysine methyltransferase 2A
LOF: Loss of Function
MLL: Mixed-lineage leukemia
NDDs: Neurodevelopmental disorders
WDSTS: Wiedemann-Steiner syndrome
