# Supplementary material for: A novel deletion mutation in KMT2A identified in a child with ID/DD and blood eosinophilia
Source: BMC Med Genet. 2019 Mar 6;20:38. doi: 10.1186/s12881-019-0776-0 (PMC6402113; doi:10.1186/s12881-019-0776-0)
Supplement: Supplementary file 1 — Table S1. Human Blood Eosinophilia-associated Mutated Genes (DOCX 26 kb) [file 12881_2019_776_MOESM1_ESM.docx]

**Table S1**. Human Blood Eosinophilia-associated Mutated Genes

| Human disorders | OMIM | Gene | Reference |
| --- | --- | --- | --- |
| Chronic granulomatous disease, with eosinophilia | 300481 | *CYBB* | [1, 2] |
| Hyper-IgE recurrent infection syndrome | 611432 | *DOCK8* | [3] |
| Idiopathic hypereosinophilic syndrome | 607685 | *PDGFRA* | [4] |
| Ichthyosis prematurity syndrome | 608649 | *SLC27A4* | [5] |
| Hypereosinophilic syndrome (HES) | 607685 | *PDGFRA* | [6] |
| Myeloproliferative disorder with eosinophilia | 131440 | *PDGFRB* | [7] |
| Total |  | 6 |  |

Chromosomal rearrangements- or CNVs-associated blood eosinophilia are not included.

**References**

1. Nguyen A, Patel K, Puck J, Dorsey M: **Longstanding Eosinophilia in a Case of Late Diagnosis Chronic Granulomatous Disease**. *J Clin Immunol* 2017, **37**(2):101-103.

2. Zhou Q, Hui X, Ying W, Hou J, Wang W, Liu D, Wang Y, Yu Y, Wang J, Sun J *et al*: **A Cohort of 169 Chronic Granulomatous Disease Patients Exposed to BCG Vaccination: a Retrospective Study from a Single Center in Shanghai, China (2004-2017)**. *J Clin Immunol* 2018, **38**(3):260-272.

3. Boztug H, Karitnig-Weiss C, Ausserer B, Renner ED, Albert MH, Sawalle-Belohradsky J, Belohradsky BH, Mann G, Horcher E, Rummele-Waibel A *et al*: **Clinical and immunological correction of DOCK8 deficiency by allogeneic hematopoietic stem cell transplantation following a reduced toxicity conditioning regimen**. *Pediatr Hematol Oncol* 2012, **29**(7):585-594.

4. Arefi M, Garcia JL, Briz MM, de Arriba F, Rodriguez JN, Martin-Nunez G, Martinez J, Lopez J, Suarez JG, Moreno MJ *et al*: **Response to imatinib mesylate in patients with hypereosinophilic syndrome**. *Int J Hematol* 2012, **96**(3):320-326.

5. Khnykin D, Ronnevig J, Johnsson M, Sitek JC, Blaas HG, Hausser I, Johansen FE, Jahnsen FL: **Ichthyosis prematurity syndrome: clinical evaluation of 17 families with a rare disorder of lipid metabolism**. *J Am Acad Dermatol* 2012, **66**(4):606-616.

6. Ogbogu PU, Bochner BS, Butterfield JH, Gleich GJ, Huss-Marp J, Kahn JE, Leiferman KM, Nutman TB, Pfab F, Ring J *et al*: **Hypereosinophilic syndrome: a multicenter, retrospective analysis of clinical characteristics and response to therapy**. *J Allergy Clin Immunol* 2009, **124**(6):1319-1325 e1313.

7. Reiter A, Gotlib J: **Myeloid neoplasms with eosinophilia**. *Blood* 2017, **129**(6):704-714.
